# Association Between Floor of Residence and Frailty in Walk-Up Buildings Among Functionally Independent Older Adults: A Cross-Sectional Study

**DOI:** 10.3390/healthcare14020162

**Published:** 2026-01-08

**Authors:** Masataka Ando, Naoto Kamide, Akie Kawamura

**Affiliations:** 1 School of Allied Health Sciences, Kitasato University, 1-15-1 Kitazato, Minami-ku, Sagamihara 252-0373, Japan; naokami@kitasato-u.ac.jp (N.K.); akie.k@kitasato-u.ac.jp (A.K.); 2 Graduate School of Medical Sciences, Kitasato University, 1-15-1 Kitazato, Minami-ku, Sagamihara 252-0373, Japan

**Keywords:** community-dwelling older adults, frailty, walk-up building, floor of residence, residential environment

## Abstract

**Background/Objectives**: Frailty has been associated with various physical, psychological, and social factors; however, the influence of the residential environment—particularly walk-up buildings without elevators—remains unclear. This study aimed to examine the association between the floor of residence and frailty among functionally independent older adults. **Methods**: A total of 793 older adults (mean age: 76.46 ± 6.29 years; 58.83% women) living in walk-up buildings without elevators and not certified as requiring long-term care participated in a questionnaire survey. Frailty was assessed using the Kihon Checklist (KCL) and the FRAIL Scale (FS). Logistic regression analyses were conducted to examine the association between floor of residence and frailty status (non-frail vs. frail), adjusting for potential confounders. Sensitivity analyses were performed using stratified models based on age group, functional status, and living conditions. **Results**: Frailty prevalence was 23.28% (KCL) and 16.88% (FS). Higher floor of residence was significantly associated with lower odds of frailty (KCL: odds ratio [OR] = 0.82, 95% confidence interval [CI]: 0.69–0.97; FS: OR = 0.80, 95% CI: 0.65–0.97). Stratified analyses showed consistent associations in subgroups including those aged ≥ 75 years, with full Instrumental Activities of Daily Living scores, non-homebound status, poor subjective economic status, and living alone (all *p* < 0.05). **Conclusions**: Living on higher floors in walk-up buildings without elevators may be protective against frailty among functionally independent older adults. While barrier-free environments are essential for those with functional decline or disabilities, moderate physical challenges such as stairs may contribute to frailty prevention in populations who maintain independence.

## 1. Introduction

Frailty is defined as a state of increased vulnerability to stressors due to age-associated declines in physiological reserve [1]. Previous studies have reported that frailty in older adults is associated with elevated risks of falls [2], disability [3], hospitalization [4], and mortality [5]. It is therefore crucial to prevent and manage frailty, not only to improve the quality of life of older adults but also to reduce social security expenditures.

Frailty is known to be influenced by a wide range of factors, including physical factors such as physical activity and exercise, psychological factors such as depression and cognitive function, and social factors such as economic status and social interaction [6]. Environmental factors have also garnered attention in recent years as potential contributors to frailty. Previous studies on environmental influences have focused on neighborhood environments surrounding the residence and suggested that aspects of the physical environment such as walkability [7,8] and green spaces [9], as well as social environments such as perceived safety from crime [7], may be associated with frailty.

Alongside broader neighborhood characteristics, increasing attention has been directed toward the indoor and adjacent residential environments, where older adults spend a significant part of their daily lives. Recent research suggests that features of the indoor built environment—such as campus-level elements unique to housing complexes and structural characteristics within and around residential buildings—may influence older adults’ physical activity and functional health [10]. One environmental feature particularly familiar to older adults in urban Japan is walk-up buildings. During the period of rapid economic growth following World War II, a large proportion of the urban housing stock was built to meet the growing demand for housing. Many of these buildings were low- to mid-rise apartment complexes without elevators, making stair use an unavoidable part of daily life. At the same time, these housing complexes have experienced a pronounced demographic shift, with the rapid aging of long-term residents. National surveys indicate that aging rates in such housing complexes are projected to reach approximately 50% by 2040, which substantially exceeds the national average of 35.3% [11]. The verticalization of housing, driven by urban economic development and planning policies, together with population aging within long-standing residential stock, may pose an emerging challenge in many countries, particularly in cities that experienced rapid urban expansion and mass housing development.

Despite growing interest in residential environments, limited evidence exists regarding the association between walk-up buildings (residential buildings without elevators, requiring frequent use of stairs in daily life) and frailty in older adults [12,13]. For older adults experiencing age-related declines in physical function, walk-up buildings can pose a barrier to going out [14]. In this context, most older adults in Japan remain functionally independent—81.7% of those aged ≥ 65 years and 68.5% of those aged ≥ 75 years have not received long-term care certification [15]—indicating that a substantial proportion of older adults retain sufficient stair-climbing capacity. From the perspective of the Person–Environment Fit model [16], it is conceivable that non-barrier-free environments such as walk-up buildings may have a protective effect against frailty for older adults who maintain relatively high levels of competence. Importantly, even within the same walk-up housing structure, the floor on which one resides determines the frequency of stair use in daily life, which may differentially influence frailty risk. Therefore, we hypothesized that, among functionally independent older adults living in walk-up buildings without elevators, residence on higher floors would be associated with a lower prevalence of frailty. The present study aims to examine the association between floor of residence and frailty among functionally independent older adults living in walk-up buildings.

## 2. Methods

### 2.1. Participants

This cross-sectional observational study was conducted among older adults (age ≥ 65 years) residing in walk-up buildings (4–5 stories) located in Sagamihara City, Kanagawa Prefecture, a suburban area near Tokyo. Participants completed a self-administered questionnaire survey. The target housing complex comprised 2526 units (2080 owner-occupied and 446 rental), with 2113 older residents, corresponding to an aging rate of 50.3%, based on the most recent demographic data available at the start of the survey (1 January 2021). The walkability of the surrounding environment was assessed using Walk Score [17], which yielded a high score of 87 out of 100, indicating that most errands can be accomplished on foot and that the area has high walkability.

The survey, which was designed as a census targeting all older residents of the housing complex, was conducted annually between 2021 and 2024. Questionnaires were distributed by post to all household, and participants were asked to return the completed forms to designated collection points. No home visits or face-to-face interviews were conducted. Although the survey was implemented multiple times, this study used a cross-sectional dataset created to avoid duplicate participants; when individuals responded more than once, only their first response was included.

The inclusion criteria were as follows: participants aged 65 years or older, able to complete the questionnaire by themselves, able to bring the completed questionnaire to designated collection points, and functionally independent in activities of daily living (ADL). Excluded were participants with ADL limitations, which were defined as being certified as requiring support (levels 1–2) or long-term care (levels 1–5) under Japan’s long-term care insurance system. These exclusion criteria were applied because the study specifically focused on examining the association between floor of residence and frailty among older adults with preserved functional competence. Additionally, given the self-administered nature of the questionnaire, individuals requiring assistance due to cognitive or functional impairment were excluded to ensure the reliability and validity of the responses.

This study was approved by the Research Ethics Committee of the School of Allied Health Sciences, Kitasato University (approval No. 2021-026-3). The purpose and content of the study were explained in a document enclosed with the questionnaire, and consent was considered to be obtained upon return of the completed questionnaire.

### 2.2. Measurements

#### 2.2.1. Frailty

Frailty was assessed using the Kihon Checklist (KCL) [18] and the FRAIL Scale (FS) [19]. The KCL consists of 25 items covering seven domains: instrumental ADL (IADL), social ADL, locomotor function, nutrition state, oral functions, outdoor activities, cognitive function, and depressive mood. Based on previous studies, participants who endorsed eight or more items were classified as frail [18]. The FS includes five items (fatigue, resistance, ambulation, illnesses, and weight loss) and is suitable for assessing physical frailty. Although the original FS defines frailty as a score of 3 or more [19], a cutoff of 2 or more has been reported as appropriate for Japanese older adults [20], and this criterion was adopted in the present study.

#### 2.2.2. Stair-Related Environmental Exposure

To assess stair-related environmental exposure, participants were asked to report their floor of residence. The buildings included in the study were not equipped with elevators, and each floor was connected by a staircase with 14 steps. Therefore, residents on higher floors were assumed to use stairs more frequently. In the analysis, floor of residence was treated as a continuous variable. However, as only about 3% of the units were located on the fifth floor and only 11 respondents (1.39%) lived on that floor, the fourth and fifth floors were combined for analysis.

#### 2.2.3. Other Variables

Additional variables included age, sex, IADL ability, homebound, medication use, dietary habits, subjective economic status, living arrangement (living alone or not), years of residence (<20/≥20 years), and housing tenure (owner-occupied/rental). IADL ability was assessed using three KCL items: “Do you go out by bus or train by yourself?”, “Do you go shopping to buy daily necessities by yourself?”, and “Do you manage your own deposits and savings at the bank?” The number of “yes” responses was used as the IADL score [21,22]. Homebound was defined as answering “no” to the KCL item “Do you go out at least once a week?” Dietary habits were assessed using the Dietary Variety Score (DVS) [23], which evaluates the frequency of consumption of 10 food groups; higher scores indicate greater dietary diversity. Subjective economic status was assessed by asking whether participants felt they had sufficient financial resources to enjoy hobbies or small luxuries.

### 2.3. Statistical Analysis

Continuous variables are presented as means ± standard deviations, and categorical variables as n (%). Differences in participant characteristics by frailty status were examined using independent *t*-tests for continuous variables and chi-square tests for categorical variables. The prevalence of frailty according to floor of residence was calculated, and trends were tested using the Cochran–Armitage trend test.

Logistic regression analyses were conducted to examine the association between floor of residence and frailty. The dependent variable was frailty status (0 = non-frail, 1 = frail) based on either the KCL or FS. The independent variable was floor of residence, and covariates included age, sex, medication use, dietary habits, subjective economic status, living arrangement, years of residence, and housing tenure. Model discrimination was evaluated using the C-statistic (areas under the receiver operating characteristic curve, AUC).

Stratified analyses were conducted according to age group (<75/≥75 years), sex, IADL score (0–2/3 points), homebound status, living arrangement, and years of residence to explore whether the association between floor of residence and frailty varied by participant characteristics. In addition, because this was a cross-sectional study and relocation bias could not be excluded—that is, the possibility that individuals who became frail may have relocated from higher floors to lower floors—interaction terms between floor of residence and age, as well as between floor of residence and years of residence, were examined. These analyses were conducted to evaluate whether the association between floor of residence and frailty varied according to age or duration of residence, and to explore the potential influence of relocation-related selection effects.

All statistical analyses were performed using R version 4.2.2 (R Foundation for Statistical Computing, Vienna, Austria), with the significance level set at 5%.

## 3. Results

A total of 935 valid responses were obtained. After excluding individuals with ADL limitations, the final analytic sample consisted of 793 functionally independent older adults (Table 1). Mean age was 76.47 ± 6.29 years and 463 (58.83%) were women. Of the overall participants, 176 (23.28%) were classified as frail based on the KCL, and 130 (16.88%) were classified as frail based on the FS. Comparisons of characteristics by frailty status revealed that for both KCL and FS, the frail group was older, had a higher proportion of individuals taking medications, and included more individuals who reported poor subjective economic status. In addition, frailty based on the KCL was associated with lower IADL score, a higher prevalence of homebound status, and lower DVS. Frailty based on the FS was significantly more prevalent among women (all *p* < 0.05).

**Figure 1:**
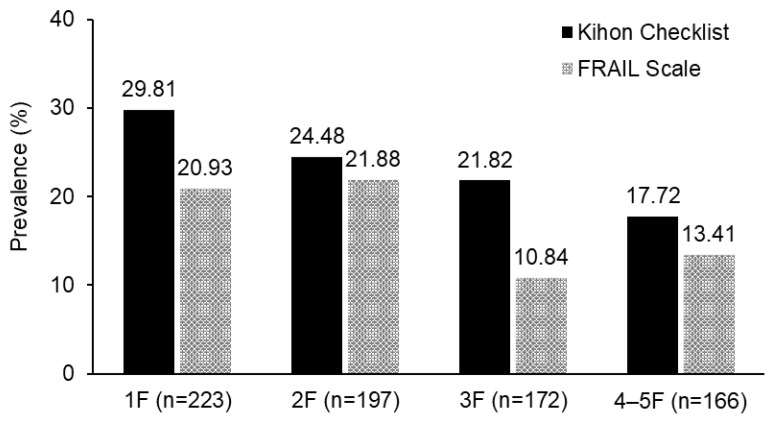
Prevalence of frailty status by floor of residence. *p*-value for trend: *p* = 0.006 (Kihon Checklist), *p* = 0.007 (FRAIL Scale). F floor.

Figure 1 shows the prevalence of frailty by floor of residence. For frailty defined by the KCL, a trend of decreasing frailty prevalence with increasing floor of residence was observed. Similarly, for frailty defined by the FS, the prevalence of frailty was lower among individuals living on the third floor or higher.

Table 2 and Table 3 present the results of logistic regression analyses adjusted for potential confounders. In the model using KCL-defined frailty as the dependent variable, higher floor of residence was significantly associated with lower odds of frailty (odds ratio [OR] = 0.82, 95% confidence interval [CI]: 0.69–0.97). Likewise, in the model using FS-defined frailty, living on a higher floor was protectively associated with frailty (OR = 0.80, 95% CI: 0.65–0.97). The models demonstrated acceptable discrimination, with AUCs of 0.71 (95% CI: 0.67–0.76) for the KCL-defined frailty model and 0.72 (95% CI: 0.67–0.77) for the FS-defined frailty model, indicating adequate ability to distinguish frail from non-frail participants.

**Figure 2:**
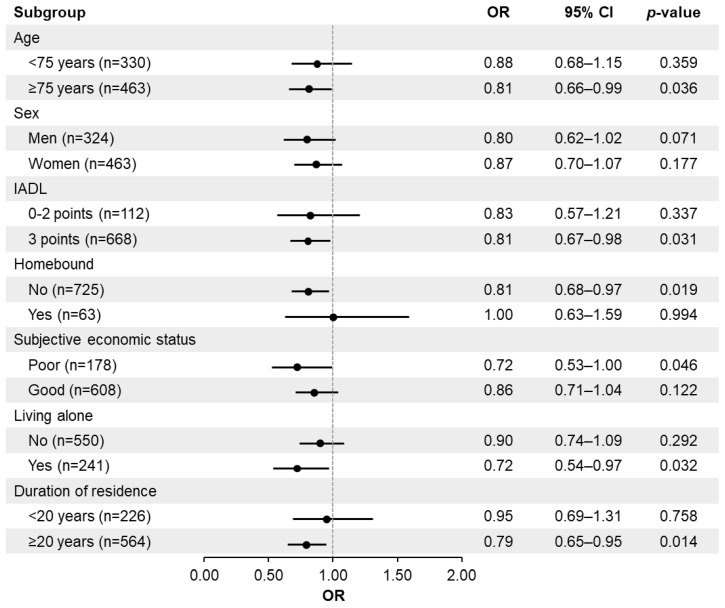
Association between comprehensive frailty status (Kihon Checklist) and floor of residence, stratified by subgroups (age group (<75/≥75 years), sex, IADL score (0–2/3 points), homebound status, living arrangement, and years of residence). Logistic regression analysis. Dependent variable: frailty status (0 = non-frail, 1 = frail). Independent variable: Floor of residence (continuous). Analyses are adjusted for age and sex. OR odds ratio, CI confidence interval, IADL instrumental activities of daily living.

**Figure 3:**
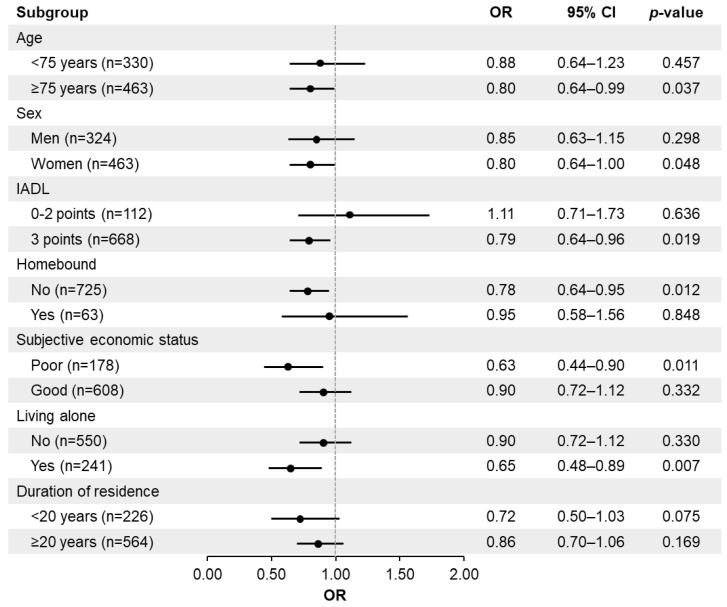
Association between physical frailty status (FRAIL Scale) and floor of residence, stratified by subgroups (age group (<75/≥75 years), sex, IADL score (0–2/3 points), homebound status, living arrangement, and years of residence). Logistic regression analysis. Dependent variable: frailty status (0 = non-frail, 1 = frail). Independent variable: Floor of residence (continuous). Analyses are adjusted for age and sex. OR odds ratio, CI confidence interval, IADL instrumental activities of daily living.

Figure 2 and Figure 3 show the results of stratified analyses. For both frailty measures (KCL and FS), higher floor of residence was significantly associated with lower prevalence of frailty among the subgroups of age ≥ 75 years, full IADL score, non-homebound, poor subjective economic status, and living alone. Additionally, in the model using KCL-defined frailty, this association was significant among individuals who had lived in their residence for 20 years or more. In the model using FS-defined frailty, the association was significant among female participants.

To examine potential relocation-related selection effects, interaction terms between floor of residence and age, as well as between floor of residence and years of residence, were included in the models. In analyses using both KCL-defined or FS-defined frailty as outcomes, none of the interaction terms were statistically significant (all *p* ≥ 0.05).

## 4. Discussion

In this cross-sectional study, we examined the association between floor of residence and frailty among functionally independent older adults living in walk-up buildings. The results of logistic regression analyses adjusted for potential confounders indicated that residing on a higher floor was protectively associated with both comprehensive frailty, as assessed by the KCL, and physical frailty, as assessed by the FS. Furthermore, stratified analyses revealed that this association varied depending on individual characteristics such as age group, IADL independence, living arrangement, frequency of going out, and subjective economic status.

A previous study suggested that living in a building without an elevator may be a risk factor for frailty among older adults [12]. However, that study included individuals with mobility limitations, who are particularly vulnerable to environments with stairs. In contrast, the present study focused exclusively on functionally independent older adults who had not been certified as requiring Japan’s long-term care insurance system. This distinction is important, because prior research has shown that the effects of environmental barriers differ according to individual functional capacity. For instance, Portegijs et al. [24] reported that environmental barriers, including stairs, were associated with restricted daily out-of-home mobility among older adults with limitations in lower extremity performance, whereas no such association was observed among those with preserved lower extremity performance. Moreover, we conducted sensitivity analyses to explore the association between floor of residence and frailty across different subgroups. This approach adds novelty to our findings and suggests that, contrary to common perceptions, stair-access housing environments may contribute to frailty prevention among older adults who maintain independence in daily activities, which is consistent with previous findings suggesting that stair-access environments are not uniformly detrimental when functional capacity is preserved.

In particular, non-barrier-free environments (those including stairs and steps) may have beneficial effects on health-related outcomes for older adults with preserved functional capacity. Tomioka et al. [25] reported that among older adults with independent IADL, living in a walk-up building was associated with a lower risk of IADL decline compared to living in a single-story or elevator-equipped building. Similarly, Mori et al. [8] found that older adults without ADL impairments or frailty who lived in physically challenging environments, such as hilly areas or places with steps, had a reduced risk of developing frailty over time. In the present study, all buildings lacked elevators, and residents of upper floors were required to use stairs daily. Such physical activity is known to support cognitive and mental health as well as physical function [26,27]. In addition, physical activity has been shown to interact positively with social interaction [28], and a dose–response relationship has been reported between stair climbing frequency and reduced mortality risk [29]. Taken together, these findings support the plausibility of our results showing that living on higher floors may help prevent both comprehensive and physical frailty.

The stratified analyses revealed significant associations between higher floor of residence and lower frailty prevalence among subgroups including those aged ≥ 75 years, those with full IADL scores, non-homebound individuals, those living alone, and those with poor subjective economic status. Physical activity tends to decline with age, and it has been reported that activity levels among adults aged ≥ 75 years fall to approximately half of those in pre-retirement age groups [30]. For this population, walk-up buildings may serve as an environmental factor that provides opportunities for daily physical activity. Furthermore, independence in IADL is associated with higher frequency of going out [14], and older adults with full IADL scores living on higher floors are likely to have more opportunities to use stairs during outings. Similarly, non-homebound individuals who go out at least once a week may also benefit from environmental factors that provide regular opportunities to use stairs in daily life. Older adults living alone and not receiving long-term care services often maintain higher functional and cognitive abilities compared to those living with others [21], suggesting that they may similarly benefit from this mechanism. For these relatively capable individuals, residing on a higher floor of a walk-up building may be particularly beneficial for frailty prevention. In addition, low socioeconomic status is a well-established social determinant of frailty and a risk factor for future disability and mortality [31]. For older adults whose access to health resources is limited due to financial constraints rather than physical limitations, living in stair-access environments may play a meaningful role in frailty prevention.

Some results varied depending on the frailty assessment tool used. Among participants classified as frail based on the KCL, a significant association between floor of residence and frailty was observed in those who had lived in their current residence for 20 years or more. Length of residence can influence social factors such as civic participation [32] and attachment to the community [33]. Long-term residence may also foster a positive perception of the living environment, including stairs, thereby reducing psychological barriers to stair use. These factors may interact to explain the protective association between floor of residence and comprehensive frailty, which includes social dimensions. In contrast, among participants classified as frail based on the FS, a significant association was found in the subgroup of women. Women experience greater declines in muscle strength and physical function compared to men [34], and our study also found a higher prevalence of physical frailty among women. Therefore, older women living on a higher floor of a walk-up building may gain additional benefit from the physical activity involved in daily stair use.

This study has several limitations. First, due to its cross-sectional design, causal relationships between floor of residence and frailty cannot be established. It is possible that individuals who became frail had already relocated from upper floors, leading to a concentration of physically robust individuals on higher floors. Although interaction analyses did not identify statistically significant effect modification by age or years of residence, relocation-related selection effects cannot be completely excluded. Accordingly, this potential for reverse causation should be considered when interpreting the findings, and future longitudinal studies are needed to clarify the directionality of this association. Second, our analysis was restricted to residents of walk-up buildings without elevators, and floor of residence was examined as an indicator of differential stair exposure within a single housing typology. Therefore, the present findings cannot directly address whether living in walk-up buildings is more or less favorable for frailty compared with living in elevator-equipped or single-story housing. Future studies that include multiple housing types will be necessary to compare the relative impacts of different residential environments on frailty. Third, the survey relied on self-administered questionnaires, and the responses were collected without home visits by researchers. Participants were required to submit their responses at designated collection points, which may have resulted in a sample biased toward relatively healthy individuals capable of traveling to those locations. However, because the analytic sample was limited to functionally independent older adults without long-term care certification, the likelihood of extensive proxy reporting is considered low. Nevertheless, informal assistance from family members cannot be completely ruled out and may have introduced some degree of measurement error. Fourth, we were unable to assess the actual frequency of stair use in detail. Although sensitivity analyses confirmed that being non-homebound and presumably using stairs daily was associated with the observed relationship between floor of residence and frailty, further investigation is needed to determine whether living on higher floors does indeed lead to increased stair use.

## 5. Conclusions

The results of this study suggest that residing on a higher floor of a walk-up building may help reduce the risk of frailty among older adults who live independently in the community. Barrier-free housing and urban environments are essential for supporting mobility among older adults with functional decline or disabilities; however, the presence of moderate physical challenges, such as stairs, may contribute to preventing frailty in certain populations, particularly those who maintain independence. In other words, uniformly removing environmental barriers such as stairs for all older adults may inadvertently accelerate the progression of frailty among those who are still physically capable. Moving forward, residential environments should be designed to accommodate the diverse needs and functional capacities of older adults.

## Figures and Tables

**Table 1:** Participant characteristics.

		Kihon Checklist			FRAIL Scale		
	Overall (n = 793)	Non-Frail (n = 580)	Frail (n = 176)	*p*-Value	Non-Frail (n = 640)	Frail (n = 130)	*p*-Value
Age (years)	76.47 ± 6.29	75.77 ± 6.00	77.93 ± 6.55	<0.001	76.00 ± 6.24	78.33 ± 6.05	<0.001
<75 years	330 (41.61)	266 (45.86)	57 (32.39)	0.002	289 (45.16)	35 (26.92)	<0.001
≥75 years	463 (58.39)	314 (54.14)	119 (67.61)		351 (54.84)	95 (73.08)	
Sex							
Men	324 (41.17)	235 (40.87)	75 (42.86)	0.704	277 (43.49)	40 (31.01)	0.012
Women	463 (58.83)	340 (59.13)	100 (57.14)		360 (56.51)	89 (68.99)	
IADL (/3 points)	2.82 ± 0.48	2.91 ± 0.31	2.53 ± 0.76	<0.001	2.84 ± 0.45	2.75 ± 0.61	0.056
0–2 points	112 (14.36)	47 (8.17)	60 (34.09)	<0.001	86 (13.63)	24 (18.46)	0.197
3 points	668 (85.64)	528 (91.83)	116 (65.91)		545 (86.37)	106 (81.54)	
Homebound							
No	725 (92.01)	548 (94.81)	147 (83.52)	<0.001	593 (92.80)	113 (87.60)	0.072
Yes	63 (7.99)	30 (5.19)	29 (16.48)		46 (7.20)	16 (12.40)	
Medications							
No	172 (22.16)	144 (25.09)	22 (12.57)	0.001	150 (23.73)	16 (12.60)	0.008
Yes	604 (77.84)	430 (74.91)	153 (87.43)		482 (76.27)	111 (87.40)	
DVS (/10 points)	4.02 ± 2.37	4.13 ± 2.34	3.47 ± 2.31	0.001	4.09 ± 2.36	3.73 ± 2.35	0.121
Subjective economic status							
Poor	178 (22.65)	105 (18.23)	65 (37.57)	<0.001	122 (19.24)	47 (36.15)	<0.001
Good	608 (77.35)	471 (81.77)	108 (62.43)		512 (80.76)	83 (63.85)	
Living alone							
No	550 (69.53)	395 (68.22)	127 (72.16)	0.370	455 (71.21)	81 (62.79)	0.073
Yes	241 (30.47)	184 (31.78)	49 (27.84)		184 (28.79)	48 (37.21)	
Duration of residence							
<20 years	226 (28.61)	168 (29.07)	50 (28.57)	0.975	183 (28.73)	40 (30.77)	0.718
≥20 years	564 (71.39)	410 (70.93)	125 (71.43)		454 (71.27)	90 (69.23)	
Housing tenure							
Owner-occupied	662 (84.01)	490 (84.92)	142 (80.68)	0.221	537 (84.17)	106 (82.81)	0.803
Rental	126 (15.99)	87 (15.08)	34 (19.32)		101 (15.83)	22 (17.19)	

Values are presented as the mean ± standard deviation or n (%). Difference among the two groups were compared by the *t*-test (for continuous variables) or the chi-square test (for categorical variables). IADL instrumental activities of daily living, DVS dietary variety score.

**Table 2:** Association between comprehensive frailty status (Kihon Checklist) and floor of residence.

	OR	95% CI	*p*-Value
Floor of residence (floors) (continuous)	0.82	0.69–0.97	0.024
Age (years) (continuous)	1.07	1.04–1.11	<0.001
Sex (women)	1.18	0.80–1.74	0.403
Medications (yes)	1.88	1.11–3.18	0.018
Dietary variety score (/10 points) (continuous)	0.86	0.79–0.94	<0.001
Subjective economic status (good)	0.37	0.24–0.56	<0.001
Living alone (yes)	0.82	0.53–1.27	0.373
Duration of residence (≥20 years)	1.04	0.66–1.64	0.868
Housing tenure (rental)	1.11	0.65–1.90	0.703

Logistic regression analysis. Dependent variable: frailty status (0 = non-frail, 1 = frail). Model discrimination was assessed using the AUC. OR odds ratio, CI confidence interval, AUC area under the receiver operating characteristic curve.

**Table 3:** Association between physical frailty status (FRAIL Scale) and floor of residence.

	OR	95% CI	*p*-Value
Floor of residence (floors) (continuous)	0.80	0.65–0.97	0.025
Age (years) (continuous)	1.07	1.03–1.11	<0.001
Sex (women)	2.16	1.36–3.42	0.001
Medications (yes)	1.66	0.92–2.99	0.093
Dietary variety score (/10 points) (continuous)	0.87	0.78–0.96	0.005
Subjective economic status (good)	0.39	0.24–0.61	<0.001
Living alone (yes)	1.47	0.92–2.32	0.104
Duration of residence (≥20 years)	0.86	0.52–1.42	0.557
Housing tenure (rental)	0.72	0.38–1.34	0.294

Logistic regression analysis. Dependent variable: frailty status (0 = non-frail, 1 = frail). Model discrimination was assessed using the AUC. OR odds ratio, CI confidence interval, AUC area under the receiver operating characteristic curve.

## Data Availability

Due to the sensitive nature of the individual-level health data used in this study and the restrictions imposed by the institutional ethics committee, the dataset cannot be made publicly available. Data may be obtained from the corresponding author upon reasonable request and with approval from the ethics committee, if required.

## Abbreviations

The following abbreviations are used in this manuscript:

ADL	Activities of daily living
AUC	Area under the receiver operating characteristic curve
CI	Confidence interval
DVS	Dietary Variety Score
FS	FRAIL Scale
IADL	Instrumental activities of daily living
KCL	Kihon Checklist
OR	Odds ratio
